# Supplementary material for: Cross-domain correspondence intensity modulation based on Bayesian-decision for remote sensing image pansharpening
Source: PLoS One. 2025 Nov 17;20(11):e0335458. doi: 10.1371/journal.pone.0335458 (PMC12622814; doi:10.1371/journal.pone.0335458)
Supplement: Attachment — Submitted filename: Response to Reviewers.docx [file pone.0335458.s003.docx]

**Response to Reviewers**

First of all, we would like to thank the reviewers for their helpful and constructive comments and suggestions. Also, we would like to thank the editor for the consideration of our manuscript. We have fully addressed the comments of the review team in the attached revised paper. We also enclose our detailed responses to the comments item-by-item and point-by-point. We mark all the changes in red in the revised paper.

**Reviewer #1:**

**The manuscript proposes a cross-domain correspondence intensity modulation framework guided by a Bayesian-decision mechanism for remote sensing pan sharpening. The goal is to enhance spatial detail transfer from PAN while preserving spectral fidelity in MS, especially under domain shifts across sensors or scenes. The authors have satisfactorily addressed all comments; acceptance is recommended.**

**Ans.** Thanks for this valuable comment.

**Reviewer #2:**

**Thank you for the author's response to my previous questions. Most of the issues have been addressed, but I still have a few minor suggestions:**

1. For Fig. 8, 9, and 10, it is recommended to include the original multispectral and panchromatic images in addition to the ground truth. This would allow readers to independently assess whether certain textural information has been lost, as the advantage of your method is not particularly evident in the provided examples. The same suggestion applies to other figures—supplementing them with original imagery would be beneficial.

**Ans.** Thanks for this valuable comment. In the revised version, to allow readers to independently assess whether certain textural information has been lost, we have included the original multispectral and panchromatic images in addition to the ground truth in Fig. 8, 9, 10, 11, and 12. Ground truth is the original multispectral image. For detailed information, please refer to the revised version.

2. In Table 5, the "Remark" column only includes comments on the PCNN. It would be helpful if remarks regarding other methods could also be added for clarity and comparison.

**Ans.** Thanks for this valuable comment. In the revised version, we have added remarks regarding other methods for clarity and comparison. The detailed information can be seen as follows:

## C. Computational Efficiency Analysis

*In this section, we handle the* *computational efficiency analysis. We calculate the average consuming time of five groups fusion images obtained in this paper for all the methods. The results were computed and listed in Table 5. From Table 5, we can find the network of PCNN requires a long time to train that results in the* *running time and resource consumption are great. IHS and BT belong to the early CS based methods. The characteristics of them are simplicity, fast, but serious spectral distortion.* *BDSD* *takes into account the image context information that results in the algorithm is relatively complex. RBDSD is* *the improved version of* *the BDSD method. So, the run time is longer than that of the the BDSD method. BDFA and the proposed method constructed* *bayesian probabilistic model to calculate the probability that each pixel in the image is selected, which has increased the complexity of the algorithm. Comparied with PCNN, the computational efficiency is high. However, the running time is longer than other latest advanced traditional pansharpening methods, which further improvements are needed in the future.*

*Table 5. The average consuming time of the comparison methods.*

| *Methods* | *Time (second)* | *remark* |
| --- | --- | --- |
| *IHS* | *0.0871* | *The early CS method had a simple algorithm* |
| *BDSD* | *0.31* | *It takes into account the context information in an image, which results in its algorithm is relatively complex.* |
| *BT* | *0.0652* | *The early CS method had a simple algorithm.* |
| *RBDSD* | *0.444* | *The improved version of the BDSD method.* |
| *BDFA* | *2.6614* | *The use of Bayesian probability models increases the complexity of the algorithm.* |
| *PCNN* | *0.4369* | - *The network training time exceeds 24 hours* |
| *Proposed* | *2.7957* | *The use of Bayesian probability models increases the complexity of the algorithm* |

3. The Conclusion section currently contains content that is more appropriate for the Discussion section. It is recommended to separate these two parts to ensure a clearer structure.

**Ans.** Thanks for this valuable comment. In the revised version, to ensure a clearer structure, we have separated the appropriate content to the discussion into the Discussion section. For detailed information, please refer to the revised version.
